# Rapid death due to pulmonary epithelioid haemangioendothelioma in several weeks: A case report

**DOI:** 10.1515/biol-2022-0073

**Published:** 2022-08-08

**Authors:** Chu Qin, Jia Hua, Xingfeng Zhu, Guochu Lu, Haoda Yu, Tao Bian

**Affiliations:** Department of Respiratory Medicine, Wuxi People’s Hospital Affiliated to Nanjing Medical University, Wuxi 214000, Jiangsu, P.R. China; Department of Nephrology, Wuxi People’s Hospital Affiliated to Nanjing Medical University, Wuxi 214000, Jiangsu, P.R. China; Department of Thoracic Surgery, Wuxi People’s Hospital Affiliated to Nanjing Medical University, Wuxi 214000, Jiangsu, P.R. China

**Keywords:** haemoptysis, pulmonary epithelioid haemangioendothelioma, lung nodules

## Abstract

A 49-year-old woman was admitted to our hospital because of haemoptysis for 6 days. This patient claimed no medical history except high blood sugar. Chest computed tomography (CT) showed infection and multiple nodules on both sides of the lung. Blood tests showed no obvious abnormalities. Tracheoscopy showed haemorrhagic discharge in the left upper lobe and an old thrombus obstructing the lumen in the anterior basal segment of the right lower lobe. Then, CT-guided percutaneous lung biopsy was performed. The pathological results suggested multiple nodular-like lesions in the submitted tissues, and tumour cells were round or short fusiform, forming a solid nest structure, visible mitosis, and a vascular cavity-like structure containing red blood cells. Immunohistochemistry revealed positive staining for Vimentin, Bcl-2, CD31, and CD34; negative staining for CD68, SMA, CR, and D2-40; and 40% Ki67+ positivity. Based on the earlier data, the patient was diagnosed with pulmonary epithelioid haemangioendothelioma. This patient did not receive any treatment for several reasons. Unfortunately, the patient died 8 weeks after diagnosis. In conclusion, we present a case featuring the rapid death due to PEH.

## 1 Manuscript novelty

Pulmonary epithelioid haemangioendothelioma (PEH) is a rare low-grade malignant neoplasm originating from vascular, the prognosis of which remains unpredictable. In the previous reports, the 5-year survival probability of PEH was 60% (range, 47–71%). One-year survival of patients without anaemia was 95% [1]. There is no specific treatment so far for PEH. PEH patients could survive from 10 to 20 years after diagnosis in most reported studies. Some asymptomatic PEH patients could get in remission in chest CT without any intervention during regular follow-up [2]. In this report, we described a rare case of PEH with a poor prognosis. To our knowledge, this case revealed the shortest survival time of PEH due to fatal haemoptysis in the current studies.

## 2 Introduction

PEH was first named intravascular bronchioloalveolar tumour by Dail et al. [3]. PEH is a rare, low-grade, malignant neoplasm originating from the vasculature. PEH mainly affects young patients, with a median age of 40 years, and is more common in women [4]. Approximately 50% of PEH cases are identified by physical examination without specific clinical manifestations [5]. PEH is usually identified by imaging, with multiple pulmonary nodules presenting as the most common imaging manifestations. There is no specific treatment thus far for PEH, and the 5-year survival rate was reported to be 60% or less [1]. In this report, we present a case featuring rapid death due to PEH at 8 weeks because of haemoptysis.

## 3 Case presentation

**Figure 1:**
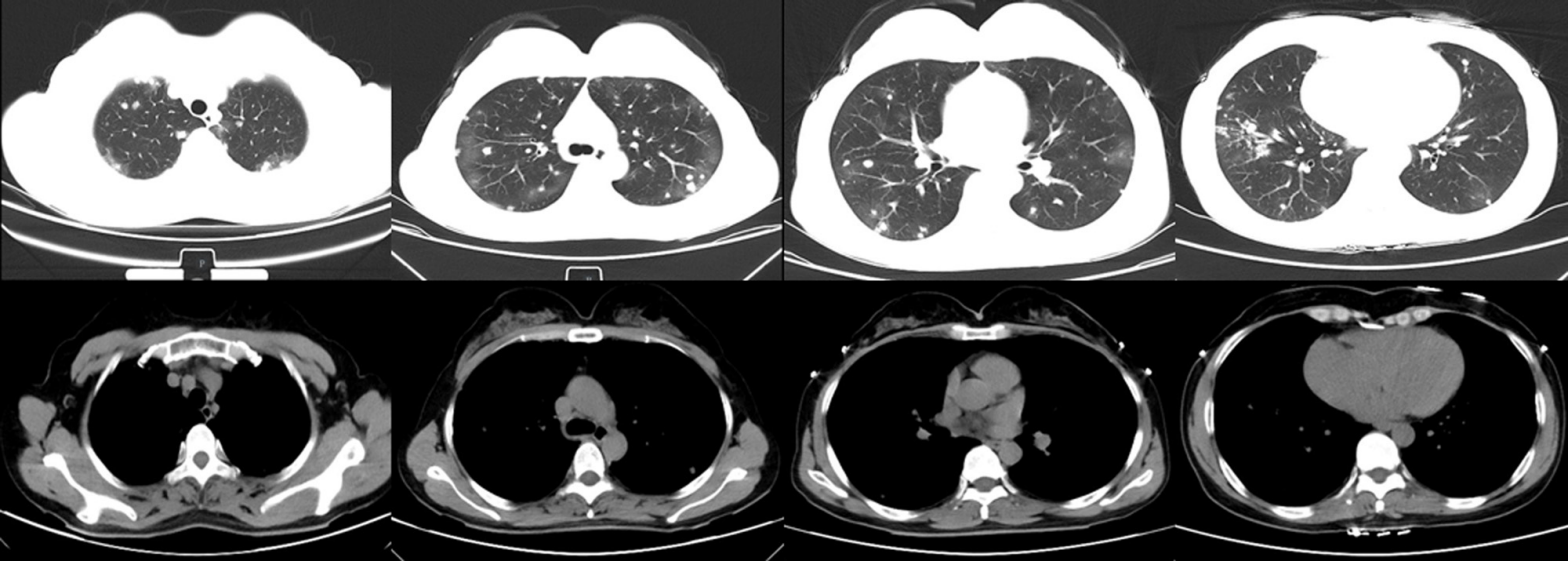
Chest CT on August 8, 2018. Chest CT showed infection and diffusely scattered nodules in both lungs.

A 49-year-old woman was admitted to our hospital complaining of haemoptysis for 6 days on August 14, 2018. She began experiencing intermittent haemoptysis accompanied by cough and itch after lunch on August 8, 2018. She claimed no chest pain and was breathless. Oral haemostatic medicine showed few therapeutic effects. Chest CT showed infection and diffusely scattered nodules in both lungs (Figure 1). She had no medical history other than hyperglycaemia, and her physical examination was unremarkable. She also denied any family history of hereditary diseases or cancer. She was admitted to our department with stable vital signs diagnosed as pulmonary infection and pulmonary nodules. Differential diagnosis was performed after admission. There were no remarkable findings on the rheumatic immune system; tumour indicators were all normal; tuberculosis and fungal infections were not indicated; and no abnormalities were found in the abdominal system, urinary system, or gynaecological ultrasonography. Thyroid ultrasonography revealed a hypoechoic mass in the bilateral neck, which was considered to be the lymph node. Bronchoscopy showed bloody secretion in the bronchus in the left upper lobe. The right inferior lobe of the base was also found to be blocked by an obsolete thrombus and could be recanalised by thrombus aspiration.

**Figure 2:**
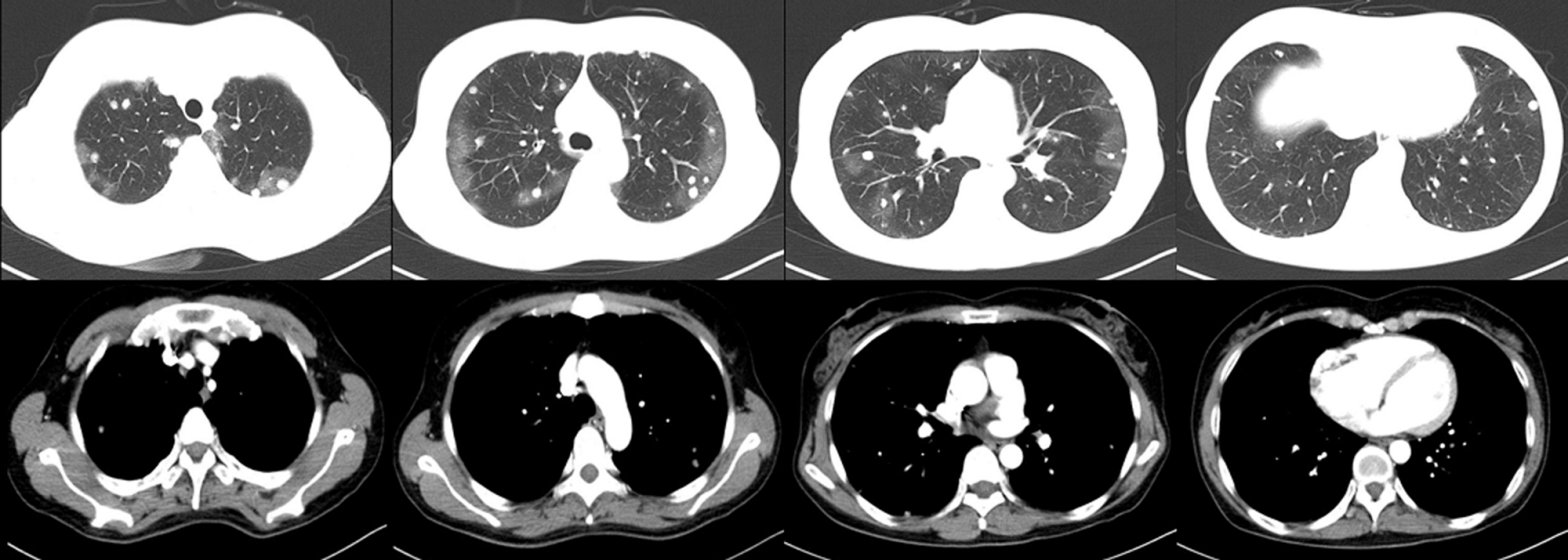
Chest CT on August 26, 2018. Re-examined chest CT showed more and larger nodules in both lungs than previously.

**Figure 3:**
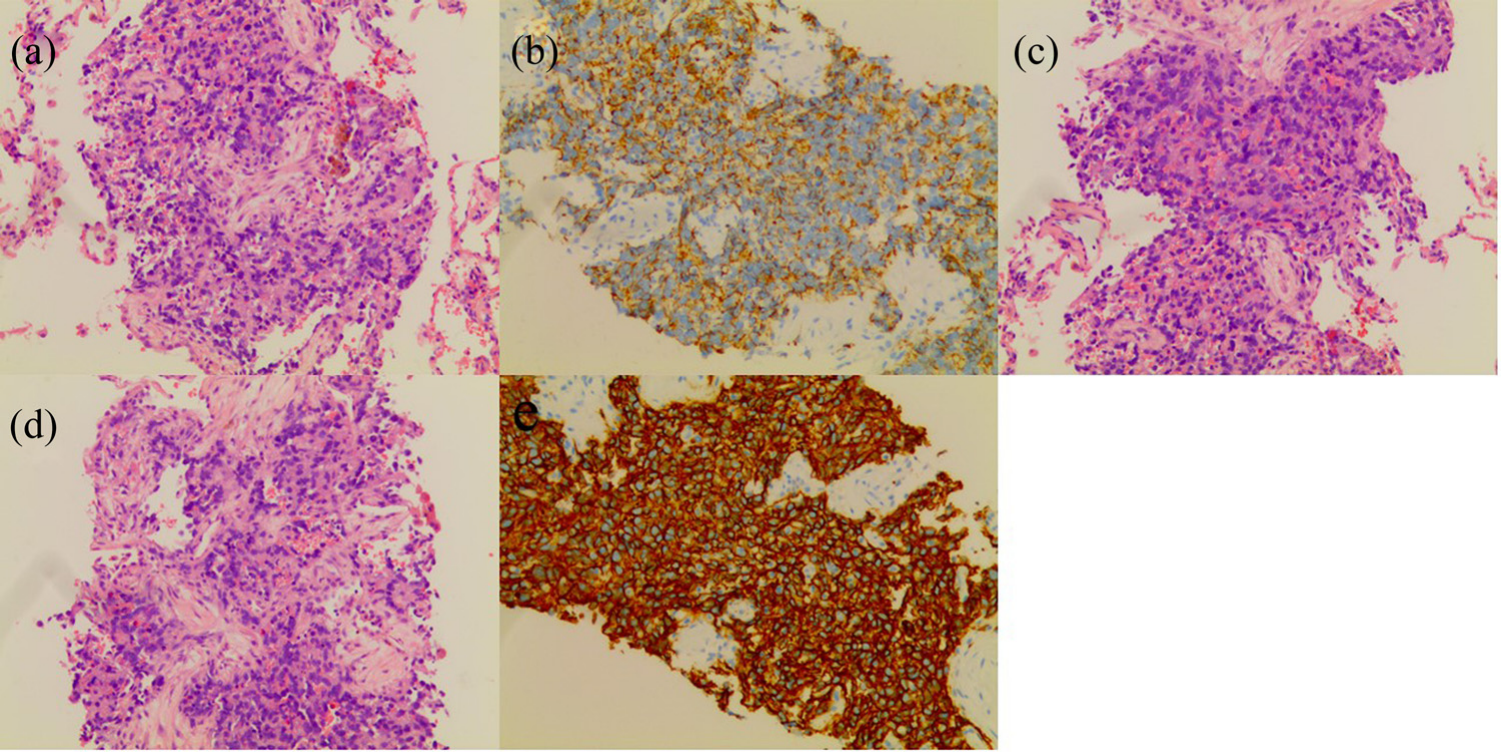
Immunohistochemical analysis. Microscopic image showed multiple nodular-like lesions in the submitted tissues, and the tumour cells were round or short fusiform, forming a solid nest structure, visible mitosis, and a vascular cavity-like structure containing red blood cells. Immunohistochemistry: positive staining for Vimentin, Bcl-2, CD31 (b), and CD34 (e); negative staining for CD68, SMA, CR, and D2–40; and 40% Ki67+ positivity.

The patient received anti-infection and haemostasis therapy. However, a re-examination by chest CT on August 24 showed more and larger nodules in both lungs than previously (Figure 2). Afterwards, a right lobe mass biopsy was performed under CT guidance. Pathology showed multiple nodular lesions and round or short spindle-shaped tumour cells, forming a solid nest structure. Nuclear divisions and vascular lacunar-like structures containing red blood cells were visible. Immunohistochemical analysis of these nodules revealed positive expression of vimentin, CD31, and CD34. This pathological examination confirmed the diagnosis of PEH (Figure 3).

**Figure 4:**
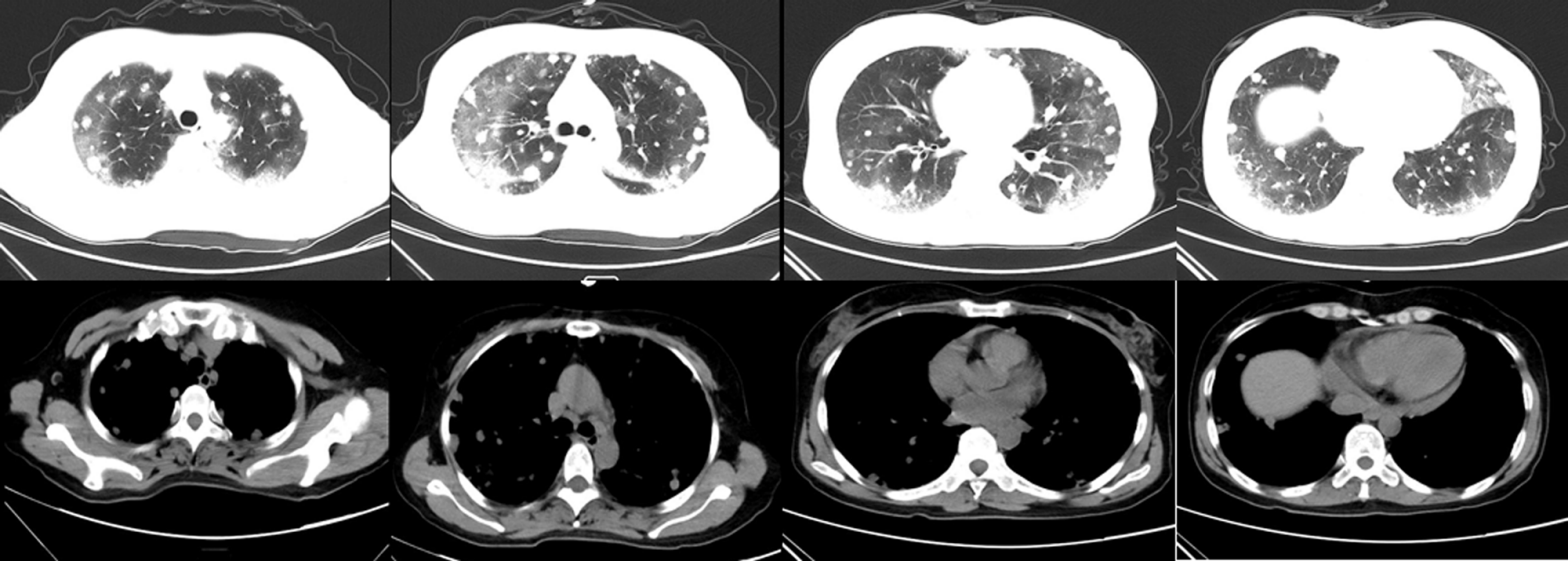
Chest CT on October 6, 2018. More exudative lesions and diffusely scattered nodules in both lungs were observed on chest CT.


Considering haemoptysis, she did not receive antiangiogenic treatment. Unfortunately, she experienced recurrent haemoptysis on October 2018. More exudative lesions and diffusely scattered nodules in both lungs were observed on chest CT (Figure 4). Active rescue failed, and this patient passed away 8 weeks after diagnosis.

**Informed consent:** Informed consent has been obtained from all individuals included in this study.
**Ethical approval:** The research related to human use has been complied with all the relevant national regulations, institutional policies and in accordance with the tenets of the Helsinki Declaration, and has been approved by the authors’ institutional review board or equivalent committee.

## 4 Discussion

Epithelioid haemangioendothelioma is a very rare, vascular, low-to-intermediate malignant tumour arising from many organ systems, including the lungs, liver, bone, and other soft tissues [6]. PEH accounts for 19% of epithelioid haemangioendotheliomas. The incidence of PEH in women is fourfold higher than that in men, and the age of patients with PEH ranges from 7 to 83, with a mean age of approximately 40 [7]. The age and sex of this patient in the current case were consistent with epidemiological characteristics. The *WWTR1/CAMTA1* gene fusion and chronic *Bartonella* infection are thought to be possible pathogenic mechanisms of PEH [7,8,9].

PEH has a poor prognosis and lacks effective diagnosis and treatment due to its low incidence and nonspecific manifestation. Most PEH patients are asymptomatic or complain of nonspecific chest problems, such as cough, chest pain, dyspnoea, and haemoptysis [10,11]. In addition, patients are often initially misdiagnosed with bronchogenic carcinoma because of multiple bilateral nodules on chest CT [12,13]. Bagan et al. reported significant factors of poor prognosis, including weight loss, anaemia, pulmonary symptoms, and pleural haemorrhagic effusions, with a median survival of less than 1 year. They also showed that patients with haemorrhagic symptoms had a statistically worse survival (haemoptysis, *p* < 0.0001; pleural effusion, *p* < 0.0001) [1]. In this case, the patient had recurrent haemoptysis. Her survival time was 8 weeks, which was the shortest among published cases to our knowledge.

There is no standard treatment for PEH thus far. The prognosis of PEH is also difficult to predict. Most reported studies have shown that PEH patients can survive from 10 to 20 years after diagnosis. Some asymptomatic PEH patients can be in remission on chest CT without any intervention during regular follow-up [2]. Surgical removal is the best choice for PEH with smaller and fewer nodules [1,14]. It was also reported that a patient with multiple bilateral lesions survived for 11 years without recurrence after the removal of 32 nodules [15]. In our case, the patient had too many nodules in both lungs to tolerate operational injury. As a result, surgery was not a considerable option for this patient. In addition to surgery, there are several adjuvant therapies for PEH, including chemotherapy, radiotherapy, and antiangiogenic therapy. Radiotherapy is suitable for patients with bone metastasis. Recently, chemotherapy was considered to have few effects on PEH [2,16]. Our patient rejected chemotherapy because of its side effects and uncertain efficacy. The vascular endothelial growth factor (VEGF) system is a new potential therapeutic target in PEH. VEGF was highly expressed in PEH and had an effect on tumour growth and invasion. In recent studies, PEH patients were relieved by antiangiogenic drugs, such as bevacizumab, apatinib, and anlotinib. Effective combination therapy was reported to be effective in some cases [17]. Considering the bleeding risk due to haemoptysis, we decided not to treat her with antiangiogenic drugs after consulting with specialists from a superior hospital. Therefore, the patient chose to wait without any treatment. However, she had recurrent massive haemoptysis, and all attempts to resuscitate her failed 8 weeks after diagnosis. In one study, symptoms such as cough, haemoptysis, chest pain, multiple unilateral nodules, and pleural effusion were all significant risk factors for PEH [18]. In this case, the patient died of suffocation caused by massive haemoptysis only 8 weeks after the diagnosis of PEH.

In conclusion, we describe a rare case of PEH with a poor prognosis. To our knowledge, this case revealed the shortest survival time of PEH due to fatal haemoptysis in the current study. For patients with PEH, both a complete assessment and a suitable treatment are necessary. Haemoptysis can lead to rapid death due to PEH. The present report may improve the clinical understanding of PEH.
